# Epidemiology of an overlapping and parallel infection of Sexually Transmitted Infections among pregnant women in North-east Ethiopia: Its implication for prevention of mother to child transmission

**DOI:** 10.1371/journal.pone.0300149

**Published:** 2024-05-20

**Authors:** Alemu Gedefie, Habtu Debash, Shambel Asmamaw, Fekadesilassie Belege Getaneh, Ermiyas Alemayehu, Asressie Molla, Altaseb Beyene Kassaw, Berhanu Kebede

**Affiliations:** 1 Department of Medical Laboratory Sciences, College of Medicine and Health Sciences, Wollo University, Dessie, Ethiopia; 2 Department of Medical Laboratory Sciences, Tropical College of Medicine, Dessie, Ethiopia; 3 Department of Pediatrics and Child Health Nursing, College of Medicine and Health Sciences, Wollo University, Dessie, Ethiopia; 4 Department of Public Health, School of Public Health, College of Medicine and Health Sciences, Wollo University, Dessie, Ethiopia; 5 Department of Biochemistry, College of Medicine and Health Sciences, Wollo University, Dessie, Ethiopia; 6 Department of Biomedical Sciences, College of Medicine and Health Sciences, Samara University, Samara, Ethiopia; Debre Markos University, ETHIOPIA

## Abstract

**Background:**

The burden of parallel and overlapping infections of Sexually Transmitted Infections (STIs), particularly HIV, syphilis, hepatitis B (HBV), and hepatitis C virus (HCV) are disproportionately higher among pregnant women globally, leading to unwanted consequences. These infections pose significant public health challenges as they can be transmitted vertically to the offspring. This study aimed to determine the sero-epidemiological patterns and predictors of STIs (HIV, syphilis, HBV, and HCV) among pregnant women attending antenatal care clinics at ten health facilities in North-eastern Ethiopia.

**Methods:**

An institution-based multi-center cross-sectional study was conducted from May to November 2022 among 422 pregnant women selected using simple random sampling technique. Semi-structured questionnaire was used to collect socio-demographic characteristics and predictor variables of STIs through face-to-face interviews. Venous blood was collected and it was tested for anti-HIV, HBsAg, anti-HCV, and anti-Treponemal antibodies using immunochromatographic test kits. Multinomial logistic regression analysis was used to identify associated factors of STIs. Variables with an adjusted odds ratio (AOR) and a p-value <0.05 were considered statistically significant.

**Results:**

The overall prevalence of STIs was 23.9% (95% CI = 20.08–28.25). The prevalence of parallel infections of HIV, hepatitis B, hepatitis C, and syphilis were 6.4%, 9%, 1.7%, and 6.9%, respectively. The overlapping infections for HIV-HBV was 4% but HIV-HCV overlapping infection wasn’t found. Increased age, tattooing, multiple sexual partners, exposure to unsafe sex, and RH status were independent factors of HBV. Likewise, increased age, rural residence, illiteracy, and tattooing were independently associated with HCV. Moreover, rural residence and a history of tattooing were independent predictors for the acquisition of HIV, whereas multiple sexual partners and RH status were found to be significant predictors of syphilis infection among pregnant women.

**Conclusion:**

The magnitude of overlapping and parallel STD infections is still continued to be a problem among pregnant women. Moreover, there were overlapping infections of HBV-HIV. Therefore, continuous screening of pregnant women for HIV, syphilis, hepatitis B, and C infections should be performed, and special attention should be given to pregnant women who have co-infections.

## 1. Introduction

Sexually Transmitted Infections (STIs) are thought to be the source of sexually transmitted diseases in both men and women with a disproportionate burden among pregnant women across countries. These infections include but are not limited to *T*. *pallidum*, hepatitis B, hepatitis C, and HIV [1]. Every day, more than a million STIs are contracted. The WHO predicted that 7.1 million new cases of syphilis would occur in 2020. An estimated 296 million people worldwide have chronic hepatitis B, which is expected to cause 820,000 deaths in 2019, primarily from hepatocellular carcinoma and cirrhosis. Numerous unfavourable delivery outcomes are linked to STIs [2, 3]. Additionally, STIs can raise the risk of HIV acquisition and have a direct influence on sexual and reproductive health globally through stigmatisation, infertility, malignancies, and pregnancy difficulties. Furthermore, low birth weight and preterm, sepsis, congenital abnormalities, stillbirth, and new born conjunctivitis can all be consequences of mother-to-child transmission of sexually transmitted infections [2].

STIs compromise quality of life, as well as sexual and reproductive health, and new born and child health. STIs during pregnancy have major consequences for the mothers and infants [4, 5]. STIs have a significant association with several adverse pregnancy and neonatal outcomes, such as spontaneous abortion, prematurity [6–8], premature rupture of membranes [9], perinatal mortality [9, 10], stillbirth and low birth weight [5, 6, 11]. Untreated STIs during pregnancy also increase the risk of miscarriage, stillbirth, neonatal deaths, prematurity, low birth weight, and congenital syphilis [10, 12, 13]. STIs increase the risk of human immunodeficiency virus (HIV) transmission, human papillomavirus infection, infertility, and physical, psychological, and social consequences [14].

To reduce the risk of maternal transmission of STIs through blood transfusion many countries have already adopted proper blood screening procedures [15, 16]. The risk of HBV transmission increases when a mother develops the infection during the third trimester of pregnancy [17]. Thus, the health status of mothers and children concerning HIV, syphilis, hepatitis B and C virus infection has become a global concern [18]. Thus, the epidemiological data for these infections are essential to health planners, and program managers and relevant for helping to develop vaccine and screening packages in antenatal care clinics.

Furthermore, to address the disproportionate burden of HIV, syphilis, hepatitis B and C infections in pregnant women which is serving as a barrier to achieving the virtual elimination of new neonatal infections, it is imperative to understand the clinical, social and geographical factors associated with this high burden of STIs. This will enable the maternal and child health programs to focus evidence-based prevention interventions on high-risk women living in the most affected geographical areas, to tip the balance in favour of a reduction in STIs. An understanding of these risk factors and the implementation of such interventions will also help to bring the country closer to the goal of eliminating vertical transmission.

The burden of overlapping STIs i.e. the presence of co-infections and parallel infection or mono-infections of HIV syphilis, HBV and HCV infections in sub-Saharan Africa (SSA) particularly in Ethiopia is high [19]. Moreover, the parallel and overlapping of HIV, syphilis, HBV and HCV infections remains a major public health problem in pregnant women. Moreover, their overlapping status is associated with the progression of liver cirrhosis which will lead to a high rate of mortality [20–22].

Furthermore, HBV and HCV co-infection has been linked with the occurrence of hepatotoxicity that is often associated with antiretroviral treatment usage [21] and remains a growing health challenge [20] and there is also inconsistency of data available about the prevalence of these infections especially in the study area. The existed evidences are also mostly reported parallel infection or mono-infections. Therefore, the burden of overlapping STIs is not well addressed and there is lack of evidence in this regard. Moreover, there is a methodological difference in assessing risk factors where the previous evidences used binary logistic regression for each STIs separately because the model can’t handle more than two values of the dependent variable but in this study, we have used multinomial logistic regression which can handle more than two values for one dependent variable. The finding from this type of analysis is more reliable. Thus, epidemiological evidence on HBV, HCV, syphilis and HIV infections among pregnant women is highly essential in Ethiopia. This study was therefore undertaken to assess the seroprevalence and potential risk factors of HBV, HCV, syphilis and HIV in pregnant women in North-east Ethiopia.

## 2. Methods and materials

### 2.1 Study setting, design, period, and population

A cross-sectional study was conducted from May to November 2022 in selected health facilities of Dessie town, North-eastern Ethiopia. Dessie is located in North Central far from the capital Addis Ababa. The town has 1 comprehensive specialised hospital, 1 General hospital, 8 health centres, 3 private hospitals, and several private clinics and specialty centres. These all-health facilities provide different health services including maternal and child health services. All pregnant women who are attending antenatal care of maternal and child health (MCH) clinics of two government hospitals, five health centres and three private hospitals and were able to give consent were included in the study. However, those who were seriously ill and unable to speak were excluded. Moreover, participants who have taken vaccines for HBV and exactly remember it were excluded.

### 2.2 Sample size determination and sampling technique

The sample size was calculated by considering the assumption of a single population proportion formula taking 8.1% of HCV [23], 95% confidence level, and 3% margin of error

$$n=\frac{{\left(z\propto /2\right)}^{2}P\;\left(1-P\right)}{d^{2}}\;n=\frac{{\left(1.96\right)}^{2}0.081\;\left(1-0.081\right)}{{\left(0.03\right)}^{2}}=318$$

Where n = sample population; P = prevalence of HCV among pregnant women (0.081); d = margin of error (0.03); Z (∝/2) = the reliability coefficient of 95% is 1.96. Finally, by adding 10% non-response rate the final sample size was 350. However, to increase the generalizability of findings we have used a total sample of 422. A simple random sampling technique was employed to select the study subjects. The flow of daily ANC attendants was used as a baseline data for the proportional allocation of pregnant women to select at random while coming into each health institution.

### 2.3 Variable

HIV, syphilis, HBV and HCV seropositivity were the dependent variables whereas socio-demographic characteristics (age, marital status, educational status, residence, occupation, and monthly income), Obstetric history (gravidity, parity, abortion, and place of delivery), risky behaviour characteristics (such as family history of hepatitis, ever heard about hepatitis virus, hospitalisation, dental extraction, blood transfusion, operation or surgery, sharing sharp materials, tattooing, ear piercing, nose piercing, venous or body piercing for treatment, multiple heterosexual partner and unprotected sex) were independent variables.

### 2.4 Data collection and laboratory data analysis

All necessary information was collected by 3 medical laboratory technologists from pregnant women attending the MCH clinic who fulfilled the inclusion criteria using semi-structured questionnaire intended for collecting sociodemographic characteristics, obstetric or gynaecological related factors and other potential risk factor data after written informed consent was obtained.

#### Blood sample collection and transportation

After obtaining informed consent, five millilitre of venous blood was collected with a plain tube labelled with a unique identification number from each study participant by a laboratory technologist. The blood sample was stored in the ice pack and if delay was unavoidable transported to the nearest health facilities and serum was separated by centrifugation at 5000 rpm for 15 minutes. Then, the serum was transported to the central laboratory of Wollo University by using a cold box and stored at -20°c until tested.

#### Anti-HIV, anti-Treponemal, HBsAg and anti-HCV antibody detection

blood specimen was tested for anti-HIV, anti-Treponemal, HBsAg and anti-HCV antibody by using the immune chromatographic technique. It was performed based on manufacturer instructions. A serum sample was added to the sample pad that was migrated to the test and control region together with colloidal gold conjugate complex and form-coloured lines in the test and control area. A coloured control band in the control region appears at the end of the test procedure regardless of the test result. This control band is the result of colloidal gold conjugate binding to immobilised particles on the membrane. The control line indicates that the colloidal gold conjugate is functional. The absence of the control band indicates that the test is invalid and will be repeated.

### 2.5 Quality assurance

To generate quality and reliable data, all quality control checks were done before, during, and after data collection. All the questions in a structured questionnaire were prepared in a clear and precise way and translated into the local language (Amharic). Pre-tests were made in 5% of the study participants in Kombolcha health centre. Then, the modification of terms and chronological arrangements were amended after pre-test. During data collection, proper categorization and coding of questionnaires and formats were made, and the collected data was checked carefully on a daily basis for completeness, accuracy, and clarity. The quality of laboratory test results was assured and standard operation procedure was used throughout all processes of sample collection and processing. The quality of test results was maintained using internal quality control (IQC) of the test kit using known negative and positive samples.

### 2.6 Data processing and analysis

Data was coded and entered into Epi-data version 3.1 and exported into a statistical package for social sciences (SPSS) version 25.0 (IMB, USA) for analysis. Descriptive statistics were computed and presented in tables and figures. The scale reliability coefficient was checked using Cronbach’s alpha (p = 0.785). The relationship between exposure variables and STIs was computed using the chi-square (χ2) test and Fisher’s exact test as required depending on the nature of the variables. Moreover, univariable and multivariable multinomial logistic regression were computed. Variables with a P value less than 0.25 in univariable analysis were subjected to multivariable multinomial logistic regression analysis Then, variables with an Adjusted odds ratio (AOR) and their p-value results <0.05 were taken as a statistically significant association.

### 2.7 Ethical considerations

Ethical approval was obtained from the College of Medicine and Health Sciences Research and Ethics Review Committee of Wollo University. Permission letter was obtained from each health facilities before the actual data collection was started. Written informed consent was obtained from each pregnant women participated in the study. Finally, pregnant women who were positive for any type of STI (HBV, HCV, HIV and/ or Syphilis) were communicated and linked to the attending physician for further management, consultation, and care.

## 3. Results

### 3.1 Socio-demographic, obstetric and risky behaviour characteristics

The mean ± standard deviation (SD) of the age of pregnant women was 27.62 ± 0.25 with 41.2% (174/422) women being found in the age group of 25–30. The proportion of STIs concerning age was increased as the age group also statistically varied (P<0.001). Of 377 (89.3%) married women 22.7% (85/377) had developed STIs. Likewise, from 306(72.5%) urban dwellers 50(16.3%) women had STIs with a statistical difference of STIs distribution across residences (P<0.001). About 278(65.9%) of pregnant women had a history of multiple pregnancies of which 76(27.3%) had STIs. The frequency distribution of STIs among women with and without a family history of any STIs was 36.1% (30/83) vs 20.9% (71/339), respectively (P = 0.007). Thirteen percent (55/422) women had a previous history of sharing sharp materials, of them 61.8% (34/55) had STIs compared with 18.3% (67/367) women who had no history of sharing sharps (P<0.001). In addition to this, 30.8% (130/422) women had tattooing and 44.6% (58) women had contracted STIs. About 38 (9.0%) and 33(7.8%) pregnant women respectively had a history of multiple sexual partners and unsafe sex (Table 1).

**Table 1:** Socio-demographic, obstetric and risky behaviour characteristics of pregnant women in North-east Ethiopia, 2022.

Variables	Category	Overall frequency	STI status		Chi-square test
			Positive	Negative	
Age group	15–19	11(2.6)	1(9.1)	10(90.9)	<0.001
	20–24	97(23.0)	9(9.3)	88(90.7)	
	25–30	174(41.2)	47(27.0)	127(73.0)	
	30–34	87(20.6)	22(25.3)	65(74.7)	
	35–39	41(9.7)	15(36.6)	26(63.4)	
	40–44	12(2.8)	7(58.3)	5(41.7)	
Religion	Muslim	206(48.8)	73(35.4)	133(64.6)	<0.001*
	Orthodox	212(50.2)	28(13.2)	184(86.8)	
	Others	4(0.9)	0(0.0)	4(100.0)	
Marital status	Single	35(8.3)	9(25.7)	26(74.3)	<0.001*
	Married	377(89.3)	85(22.7)	292(77.5)	
	Divorced	3(0.7)	0(0.0)	3(100.0)	
	Widowed	7(1.7)	7(100.0)	0(0.0	
Residence	Rural	116(27.5)	51(44.0)	65(56.0)	<0.001
	Urban	306(72.5)	50(16.3)	256(83.7)	
Educational status	Unable to read and write	118(28.0)	43(36.4)	75(63.6)	0.001
	Primary education	66 (15.6)	8(12.1)	58(87.9)	
	Secondary education	91(21.6)	16(17.6)	75(82.4)	
	College and above	147(34.8)	34(23.1)	113(76.9)	
Occupation	Student	68(16.1)	18(26.5)	50(73.5)	0.004*
	Gov’t employee	85(20.1)	17(20.0)	68(80.0)	
	Un employed	53(12.3)	22(41.5)	31(58.5)	
	House wife	194(46)	44(22.7)	150(77.3)	
	Farmer	16(3.8)	0(0.0)	16(100.0)	
	Other	6(1.4)	0(0.0)	6(100.0)	
Multiple pregnancy	Yes	278(65.9)	76(27.3)	202(72.7)	0.023
	No	144(34.1)	25(17.4)	119(82.6)	
Place of delivery (n = 278)	House	36(8.5)	17(47.2)	19(52.8)	0.002
	Health facility	242(57.3)	59(24.4)	183(75.6)	
Parity	1	141(33.4)	25(17.7)	116(82.3)	0.006*
	2–4	261(61.8)	75(28.7)	1186(71.3)	
	≥5	20(4.7)	1(5.0)	19(95.0)	
History of abortion	Yes	48(11.4)	19(39.6)	29(60.4)	0.007
	No	374(88.6)	82(21.5)	292(78.1)	
Family history of any STIs	Yes	83(19.7)	30(36.1)	53(63.9)	0.004
	No	339(80.3)	71(20.9)	268(79.1)	
History of dialysis	Yes	10(2.4)	0(0.0)	10(100.0)	0.156*
	No	412(97.6)	101(24.5)	311(75.5)	
Hospitalisation	Yes	71(16.8)	15(21.1)	56(78.9)	0.543
	No	351(93.2)	86(24.5)	265(75.5)	
Tooth extraction	Yes	98(23.2)	14(14.3)	84(85.7)	0.011
	No	324(76.8)	87(26.9)	237(73.1)	
Transfusion	Yes	19(4.5)	7(36.8)	12(63.2)	0.177
	No	403(95.5)	94(23.3)	309(76.7)	
Surgery	Yes	24(5.7)	6(25.0)	18(75.0)	0.900
	No	398(94.3)	95(23.9)	303(76.1)	
Sharing sharp materials	Yes	55(13.0)	34(61.8)	21(38.2)	<0.001
	No	367(87.0)	67(18.3)	300(81.7)	
Tattooing	Yes	130(30.8)	58(44.6)	72(55.4)	<0.001
	No	292(69.2)	43(14.7)	249(85.3)	
Nose-ear piercing	Yes	401(95.0)	94(23.4)	307(76.6)	0.300
	No	21(5.0)	7(33.3)	14(66.7)	
Venous cutting	Yes	36(8.5)	14(38.9)	22(61.1)	0.028
	No	386(91.5)	87(22.5)	299(77.5)	
Multiple sexual partner	Yes	38(9.0)	8(21.1)	30(78.9)	0.663
	No	384(91.0)	93(24.2)	291(75.8)	
Unsafe sex	Yes	33(7.8)	14(42.4)	19(57.6)	0.010
	No	389(92.2)	87(22.4)	302(77.6)	
ABO blood group type	A	127(30.1)	38(29.9)	89(70.1)	0.171
	B	80(18.9)	15(18.8)	65(81.3)	
	AB	59(14.0)	16(27.1)	43(72.9)	
	O	156(37.0)	32(20.5)	(79.5)	
RH status	RH+	341(80.8)	63(18.5)	278(81.5)	<0.001
	RH-	81(19.2)	38(46.9)	43(53.1)	

*: Fisher’s exact test was used since assumption of chi-square (χ2) test was violated; STIs: Sexually Transmitted Infections

### 3.2 Overlapping and parallel infections of STIs

**Fig 1:**
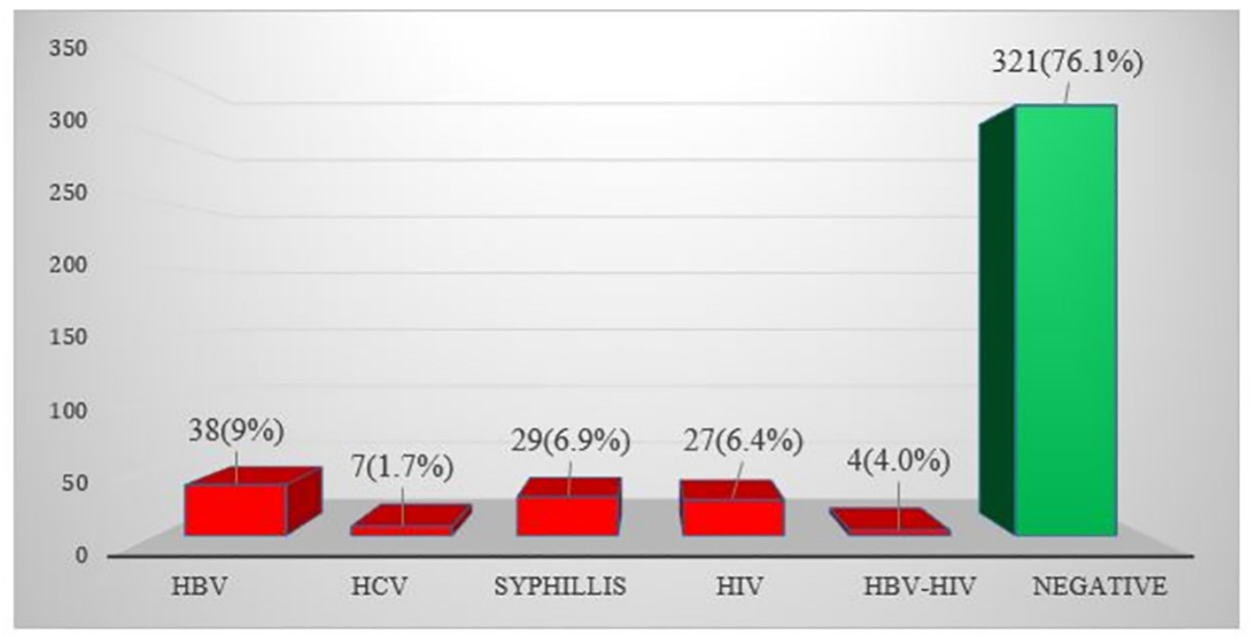
Distribution of STIs among pregnant women in North-east Ethiopia, 2022.

The overall prevalence of STIs among pregnant women was 23.9% (95% CI = 20.08–28.25). An overlapping infection of HBV-HIV co-infection was detected in 4/101(4%; 95% CI = 2.3–5.6) of pregnant women while the rest were parallel infections with an overall prevalence of 9% (6.6–12.1), 6.9% (4.8–9.7), 6.4% (4.4–9.2) and 1.7% (0.94–3.4) respective distribution of HBV, Syphilis, HIV and HCV infections Fig 1.

The highest frequency of HBV among urban dwellers and illiterate women was 60.5% (23/38) and 47.4% (18/38), respectively. Moreover, the predominance of HBV infection among pregnant women was observed among those who have a history of tattooing (57.9%), nose-ear piercing (97.4%), multiple sexual partners (86.8%), unsafe sex (71.1%) and among 76.3% of RH positive women. Furthermore, all hepatitis C virus-positive women had a history of nose-ear piercing, unsafe sex, multiple sexual partners, and home delivery as well as all of these infected women were rural residents and RH positives without a history of abortion. Among 29 syphilis-positive women, all of them were from rural areas and had a history of institutional delivery as well as 2–4 parity status. In addition, 26 of them (89.7%) were RH positives and had a history of multiple sexual partners. On the contrary, all HIV-positive women had no history of abortion, hospitalisation, transfusion and surgery; however, all of these HIV-positives had a history of nose-ear piercing, multiple sexual partners and unsafe sex (Table 2).

**Table 2:** Distribution of sexually transmitted infections among pregnant women in North-east Ethiopia, 2022.

Variables	Category	Type of sexually transmitted infections					Overall (N = 422)	Chi-square test
		HBV	HCV	Syphilis	HIV	Total		
Age group	15–19	1(2.6)	0(0.0)	0(0.0)	0(0.0)	1(9.1)	11(2.6)	<0.001
	20–24	3(7.9)	0(0.0)	0(0.0)	6(22.2)	9(9.3)	97(23.0)	
	25–30	20(52.6)	0(0.0)	13(44.8)	14(51.9)	47(27.0)	174(41.2)	
	30–34	6(15.8)	1(14.3)	16(55.2)	0(0.0)	22(25.3)	87(20.6)	
	35–39	1(2.6)	5(71.4)	0(0.0)	7(25.9)	15(36.6)	41(9.7)	
	40–44	7(18.4)	1(14.3)	0(0.0)	0(0.0)	7(58.3)	12(2.8)	
Religion	Muslim	26(68.4)	5(71.4)	20(100)	20(74.1)	73(35.4)	206(48.8)	<0.001
	Orthodox	12(31.6)	2(28.6)	0(0.0)	7(25.9)	28(13.2)	212(50.2)	
	Others	0(0.0)	0(0.0)	0(0.0)	0(0.0)	0(0.0)	4(0.9)	
Marital status	Single	3(7.9)	1(14.3)	0(0.0)	6(22.2)	9(25.7)	35(8.3)	<0.001
	Married	31(81.6)	4(57.1)	26(89.7)	21(77.8)	85(22.7)	377(89.3)	
	Divorced	0(0.0)	1(14.3)	0(0.0)	0(0.0)	0(0.0)	3(0.7)	
	Widowed	4(10.5)	1(14.3)	3(10.3)	0(0.0)	7(100.0)	7(1.7)	
Residence	Rural	15(39.5)	7(100)	29(100)	6(22.2)	51(44.0)	116(27.5)	<0.001
	Urban	23(60.5)	0(0.0)	0(0.0)	21(77.8)	50(16.3)	306(72.5)	
Educational status	Illiterate	16(42.1)	4(57.1)	20(69)	0(0.0)	43(36.4)	118(28.0)	<0.001
	Primary	2(5.3)	1(14.3)	6(20.7)	0(0.0)	8(12.1)	66 (15.6)	
	Secondary	6(15.8)	1(14.3)	3(10.3)	7(25.9)	16(17.6)	91(21.6)	
	College and above	14(36.8)	1(14.3)	0(0.0)	20(74.1)	34(23.1)	147(34.8)	
Occupation	Student	5(13.2)	0(0.0)	0(0.0)	13(48.1)	18(26.5)	68(16.1)	<0.001
	Gov’t employee	10(26.3)	1(14.3)	0(0.0)	7(25.9)	17(20.0)	85(20.1)	
	Un employee	5(13.2)	3(42.9)	17(58.6)	0(0.0)	22(41.5)	53(12.3)	
	House wife	18(47.4)	3(42.9)	12(41.4)	7(25.9)	44(22.7)	194(46)	
	Farmer	0(0.0)	0(0.0)	0(0.0)	0(0.0)	0(0.0)	16(3.8)	
	Other	0(0.0)	0(0.0)	0(0.0)	0(0.0)	0(0.0)	6(1.4)	
Multiple pregnancy	Yes	5(13.2)	5(71.4)	0(0.0)	20(74.1)	76(27.3)	278(65.9)	0.001
	No	33(86.8)	2(28.6)	29(100)	7(25.9)	25(17.4)	144(34.1)	
Place of delivery (n = 278)	House	10(30.3)	7(100)	0(0.0)	20(74.1)	17(47.2)	36(8.5)	<0.001
	Health facility	23(69.7)	0(0.0)	29(100)	7(25.9)	59(24.4)	242(57.3)	
Parity	1	5(13.2)	0(0.0)	0(0.0)	20(74.1)	25(17.7)	141(33.4)	<0.001
	2–4	32(84.2)	7(100)	29(100)	7(25.9)	75(28.7)	261(61.8)	
	≥5	1(2.6)	0(0.0)	0(0.0)	0(0.0)	1(5.0)	20(4.7)	
History of abortion	Yes	10(26.3)	0(0.0)	9(31.9)	0(0.0)	19(39.6)	48(11.4)	<0.001
	No	28(73.7)	7(100)	20(69)	27(100)	82(21.5)	374(88.6)	
Family history of any STIs	Yes	6(15.8)	0(0.0)	17(58.6)	7(25.9)	30(36.1)	83(19.7)	<0.001
	No	32(84.2)	7(100)	12(41.4)	20(74.1)	71(20.9)	339(80.3)	
Hospitalisation	Yes	9(23.7)	0(0.0)	6(20.7)	0(0.0)	15(21.1)	71(16.8)	0.073
	No	29(76.3)	7(100)	23(79.3)	27(100)	86(24.5)	351(93.2)	
Tooth extraction	Yes	4(10.5)	0(0.0)	3(10.3)	7(25.9)	14(14.3)	98(23.2)	0.042
	No	34(89.5)	7(100)	26(89.7)	20(74.1)	87(26.9)	324(76.8)	
Transfusion	Yes	1(2.6)	0(0.0)	6(20.7)	0(0.0)	7(36.8)	19(4.5)	<0.001
	No	37(97.4)	7(100)	23(79.3)	27(100)	94(23.3)	403(95.5)	
Surgery	Yes	0(0.0)	0(0.0)	6(20.7)	0(0.0)	6(25.0)	24(5.7)	0.002
	No	38(100)	7(100)	23(79.3)	27(100)	95(23.9)	398(94.3)	
Sharing sharp materials	Yes	10(26.3)	5(71.4)	17(58.6)	7(25.9)	34(61.8)	55(13.0)	<0.001
	No	28(73.7)	2(28.6)	12(41.4)	20(74.1)	67(18.3)	367(87.0)	
Tattooing	Yes	22(57.9)	4(57.1)	23(79.3)	13(48.1)	58(44.6)	130(30.8)	<0.001
	No	16(42.1)	3(42.8)	6(20.7)	14(51.9)	43(14.7)	292(69.2)	
Nose-ear piercing	Yes	37(97.4)	7(100)	23(79.3)	27(100)	94(23.4)	401(95.0)	0.001
	No	1(2.6)	0(0.0)	6(20.3)	0(0.0)	7(33.3)	21(5.0)	
Venous cutting	Yes	4(10.5)	5(71.4)	10(34.5)	5(18.5)	14(38.9)	36(8.5)	<0.001
	No	34(89.5)	2(28.6)	19(65.5)	22(81.5)	87(22.5)	386(91.5)	
Multiple sexual partner	Yes	33(86.8)	7(100)	26(89.7)	27(100)	93(24.2)	384(91.0)	<0.001
	No	5(13.2)	0(0.0)	3(10.3)	0(0.0)	8(21.1)	38(9.0)	
Unsafe sex	Yes	27(71.1)	7(100)	26(89.3)	27(100)	87(22.4)	389(92.2)	<0.001
	No	11(28.9)	0(0.0)	3(10.3)	0(0.0)	14(42.4)	33(7.8)	
ABO blood group type	A	5(13.2)	3(42.9)	13(44.8)	13(48.1)	38(29.9)	127(30.1)	0.101
	B	8(21.1)	1(14.3)	7(24.1)	0(0.0)	15(18.8)	80(18.9)	
	AB	7(18.4)	1(14.3)	9(31.0)	0(0.0)	16(27.1)	59(14.0)	
	O	18(47.4)	2(28.6)	0(0.0)	14(51.9)	32(20.5)	156(37.0)	
RH status	RH+	29(76.3)	7(100)	26(89.7)	14(51.9)	63(18.5)	341(80.8)	<0.001
	RH-	9(23.7)	0(0.0)	3(10.3)	13(48.1)	38(46.9)	81(19.2)	

### 3.3 Factors associated with STIs among pregnant women

Since the assumption of binary logistic regression was violated i.e the dependent variable has more than two values multinomial logistic regression was selected for assessing the predictors of STIs. After bivariable multinomial logistic regression was computed variables with a p<0.25 were subjected to multivariable multinomial logistic regression analysis. The goodness of fit test of the model was assessed by the Hosmer-Lemeshow test (p = 0.349) and the reliability coefficient using Cronbach’s alpha was 0.785.

Finally, after controlling confounding, the multivariable multinomial logistic regression analysis showed that the following factors were independently associated with different types of STIs. HBV infection was independently predicted by increased age (AOR = 1.19; 95% CI = 1.03–1.38; p<0.05), history of tattooing (AOR = 2.56; 95% CI = 1.01–6.42; p<0.05), multiple sexual partner (AOR = 6.12; 95% CI = 2.3–9.26; p<0.05), exposure of unsafe-sex (AOR = 7.35; 95% CI = 4.04–18.2; p<0.01) and being RH positive (AOR = 3.16, 95% CI = 1.58–9.61; p<0.001). Moreover, HCV was independently associated with increased age of women (AOR = 1.76; 95% CI = 1.46–2.11; p<0.001), rural residence (AOR = 7.93; 95% CI = 3.28–17.4, p<0.001), educational status being illiterate (AOR = 6.75; 95% CI = 1.26–36.04, p<0.05) and history of tattooing (AOR = 1.35; 95% CI = 1.03–2.68; p<0.05). Rural residence (AOR = 0.48; 95% CI = 0.39–0.74; p<0.01) and history of tattooing (AOR = 7.61; 95% CI = 3.03–19.11; p<0.01) were independent predictors for the acquisition of HIV whereas multiple sexual partner (AOR = 8.23; 95% CI = 4.12–12.94; p<0.001) and RH status (AOR = 8.29; 95% CI = 3.13–17.21; p<0.01) were found as a significant predictors of syphilis infection among pregnant women (Table 3).

**Table 3:** Multivariable multinomial logistic regression analysis of factors associated with STIs among pregnant women in North-east Ethiopia, 2022.

Variables	Category	Sexually transmitted infections			
		HBV	HCV	HIV	Syphilis
		AOR (95% CI)	AOR (95% CI)	AOR (95% CI)	AOR (95% CI)
Age		1.19(1.03–1.38) *	1.76(1.46–2.11) ***	0.97(0.79–1.20)	0.98(0.79–1.20)
Gravidity		0.214(0.013–3.6)	1.28(0.056–28.99)	0.29(0.08–10.19)	0.36(0.01–10.85)
Parity		3.07(0.17–5.53)	0.13(0.005–3.22)	4.48(0.12–16.7)	1.31(0.04–4.09)
Residence	Rural	1.24(0.31–5.02)	7.93(3.28–17.4) ***	0.48(0.39–0.74) **	0.66(0.07–6.52)
	Urban	1	1	1	1
Educational status	Illiterate	1.19(0.36–3.96)	6.75(1.26–36.04) *	0.65(0.08–5.69)	0.28(0.05–1.65)
	Primary	0.56(0.12–2.55)	0.26(0.03–1.99)	1.87(0.3–10.65)	0.5(0.08–2.97)
	Secondary	0.65(0.19–2.2)	1.25(0.23–6.71)	0.78(0.11–5.68)	1.26(0.44–3.38)
	Tertiary	1	1	1	1
Abortion	Yes	19.1(7.67–47.45)	1.75(1.29–2.36)	0.71(0.02–2.35)	0.27(0.06–1.12)
	No	1	1	1	1
Family history of STI	Yes	0.89(0.24–3.1)	0.62(0.12–3.14)	0.6(0.12–3.14)	2.45(0.94–6.39)
	No	1	1	1	1
Dialysis	Yes	0.42(0.017–10.63)	0.49(0.005–4.95)	0.49(0.05–4.95)	0.72(0.01–4.26)
	No	1	1	1	1
Hospitalisation	Yes	2.45(0.83–7.19)	0.34(0.05–2.15)	0.34(0.06–2.15)	0.57(0.09–3.34)
	No	1	1	1	1
Tooth extraction	Yes	0.22(0.06–0.77)	0.23(0.05–1.04)	0.29(0.05–1.04)	0.36(0.1–1.45)
	No	1	1	1	1
Transfusion	Yes	0.73(0.05–10.12)	0.68(0.03–1.71)	0.68(0.03–1.71)	0.32(0.01–11.79)
	No	1	1	1	1
Surgery	Yes	0.16(0.009–2.80)	3.92(0.06–5.42)	3.93(0.06–24.26)	2.31(0.06–8.31)
	No	1	1	1	1
Sharp	Yes	1.01(0.026–39.41)	0.08(0.001–5.965)	0.79(0.139–5.96)	0.31(0.02–0.79)
	No	1	1	1	1
Tattooing	Yes	2.56(1.01–6.42) *	1.35(1.03–2.68) *	7.61(3.03–19.11) **	0.13(0.5–1.68)
	No	1	1	1	1.2
Nose-ear piercing	Yes	0.61(0.08–4.48)	4.79(0.12–18.79)	4.79(0.12–18.72)	.2(0.04–3.66)
	No	1	1	1	1
Venous cutting	Yes	0.71(0.12–4.16)	0.46(0.06–3.57)	0.46(0.58–3.57)	0.28(0.01–7.07)
	No	1	1	1	1
MSP	Yes	6.12(2.3–9.26) *	0.99(0.09–10.67)	0.99(0.09–10.67)	8.23(4.12–12.94) ***
	No	**1**	1	1	1
Unsafe-sex	Yes	7.35(4.04–18.2) **	1.56(0.11–21.5)	1.56(0.11–21.5)	0.3(0.05–1.79)
	No	1	1	1	1
RH status	RH+	3.16(1.58–9.61) ***	0.96(0.19–7.67)	0.84(0.139–2.96)	8.29(3.13–17.21) **
	RH-	1	1	1	1

## 4. Discussion

STIs are still continued as a public problem requiring sustainable prevention strategy and policy. The present multi-center sero-epidemiologic survey had confirmed that pregnant women had still faced a catastrophic problem which might lead to unwanted pregnancy outcome and death. In this study, the overall prevalence of STIs (HIV and /or HBV and /or HCV and /or Syphilis) was 23.9%, which implies that nearly one in every four pregnant women might develop STIs. This figure revealed that STIs are a significant cause of morbidity and mortality among pregnant women particularly in resource limited countries like Ethiopia. Thus, collaborative inter-sectoral actions should be performed to reduce the burden of these STIs. If we exert our maximum effort for reduction of the rapidly growing burden of STIs, we can limit the vertical transmission of infections to the coming generations unless and otherwise the rate of parallel and overlapping infections of HIV, HBV, HCV and syphilis will aggravates more than the expected endemic state.

The overlapping infection of STIs in this study was found in 4% of pregnant women with the co-infection of HBV-HIV. This finding was in agreement with 2.7% co-infection reported from Nigeria [24] but it was higher than 1.1% of another Nigeria study [25]. The shared way of transmission of the viruses (unsafe sexual intercourse, use of unsterilized sharp objects, blood transmission and vertical transmission from mother to fetus among others) might contribute for the overlapping infection. On behalf of this, the extent of overlapping infection implies the risk of worsen health problems and pregnancy outcomes due to the dual effect of the two viral pathogenesis. In addition, it also signifies the need to control monitoring of pregnant women who had overlapping infections to limit the adverse outcomes and vertical transmissions.

On the other hand, the highest rate of parallel infections was found in this study. The prevalence of HBV was 9% which was in agreement with 10.9% [26] and 7.2% [27] in Southern Ethiopia, 8% in Mali [28] and 6.78% in Nigeria [29] but also higher than previous Ethiopian reports of 3.5% [30] and 6.0% [31]. According to the global magnitude of HBsAg, the present finding is clustered as high (⩾8%). This difference might be attributed to differences in time period related with hidden transmission of HBV, health information dissemination programs, socio-economic status of participants as well as risky sexual behaviours all of which increases the rate of infections.

The magnitude of HCV infection was 1.7% which was in agreement with the 2.6% anti-HCV reported from Nigeria [32] but lower than 8.1% of anti-HCV in Ethiopia [23] and 6.0% from Nigeria [33]. The existing discrepancy might be associated with methodological differences like sample size, risky behaviour and socioeconomic status of participants as well as the laboratory diagnostic approach. The utilisation of enzyme linked immunosorbent assay (ELISA) in Western Ethiopia study [23] might contribute to the highest yield of anti-HCV cases than the immunochromatographic test method we used. Therefore, the difference in sensitivity of laboratory diagnostic methods might contribute to the existing discrepancy.

The prevalence of HIV was 6.4% which implies that nearly six women in every hundred pregnant women had developed HIV infection which is supported by previous evidence reporting women in the developing world are at higher risk of HIV infection in relation to biological attributes. The female reproductive tract possesses various features that render it vulnerable to viral colonization, infection, and systemic dissemination. The high affinity of Langerhans’ cells of the cervix and Vaginal inflammation facilitating the entry of HIV [34]. These events result in localized adaptations that could potentially enable secondary microbial infection. The virus interacts with resident cells of the innate immune system, adaptive immune system, and mucosal-associated lymphoid tissue (MALT) during the development of infection, as well as typical cells of the female genital tract, such as epithelial cells [35]. In addition, the finding of the current study was agreed with the finding of systematic review and meta-analysis report of 5.74% of HIV in Ethiopia reported in 2018 [36]. Despite the fact that the representativeness of sample size differences between the systematic review and meta-analysis and this study, the present study implies a stable transmission and /or endemicity of HIV in Ethiopia.

The overall syphilis positivity rate in this study was 6.9% which was lower than 11.8% of positivity reported from pregnant women from camps in Western Ethiopia [37]. Behaviour change following aggressive reproductive health education campaigns and access to health care service including information education communication on reproductive health, variations sexual behaviour, improvement in health seeking behaviour to treatment of syphilis, and the variation in type of population being studied might contribute for a decreased magnitude of syphilis in this study.

The magnitude of HIV, HBV and HCV among pregnant women who had a history of tattooing was significantly higher compared to their counterparts. This could be due to the fact that the triple viral infections (HIV, HBV and HCV) are STIs and that have shared way of transmission, which can be transmitted through ineffective utilisation of infection protective tools, sharing and repeated use of unsterilized instruments for the tattooing procedure and technical errors or faults that unskilled and/or traditional person commit during tattooing procedure [38].

Furthermore, pregnant women who had a history of multiple heterosexual partners had a higher rate of HBV and syphilis infection than their counterparts. This might be due to the fact that the transmission of STIs particularly HBV and syphilis infection increases with the number of sexual partners as well as the duration of sexual activity. Based on previous evidence, the risk of acquiring STIs among adolescents having sex with multiple partners is high [39, 40]. Moreover, pregnant women who had a history of unprotected sex had a higher rate of HBV acquisition than those who committed protected sex. This might be because risky sexual behaviours can increase the rate of infections as they are the means of viral transmission.

The magnitude of viral hepatitis infection (HBV and HCV) was disproportionately increased as the age of pregnant women increases. An increased risk of infection with age might be linked with the proxy for lifetime exposure [41] because the cumulative risk of viral hepatitis infection is associated with sexual and percutaneous exposures over time.

The risk of HCV was significantly higher in rural than urban dwellers. The reason behind might be the level of awareness in rural settings is low and access to mass media and social networks are limited in rural settings. In addition, in relation to low health care access, health care seeking behaviour and level of awareness on the transmission and prevention methods of STI, the vulnerability of rural dwellers might be increased. For example, there is a lack of access to condoms in rural Ethiopia and also the cultural habits to request it is extremely low. So, this will exacerbate the transmission of STIs (HCV). On the other hand, the risk of HIV was significantly higher in urban than rural dwellers. Majorities (72.5%) of pregnant women in this study were from urban residents which might contribute to increased prevalence of HIV in urban settings. In addition, urban women are more likely to engage in sex work due to harmful sexual behavior, transactional sex work, poverty, and limited economic opportunities. Additionally, alcohol and substance use are common in urban settings such as bars, clubs, and parties, which may contribute to higher rates of HIV infection [42, 43].

The prevalence of HCV was negatively associated with educational status of pregnant women where women whose educational status of illiterate was 6.7 times more likely to acquire HCV infection than those who have tertiary level of education. This might be explained by the fact that the level of knowledge about HCV transmission and prevention mechanisms might be inadequate in women with low educational levels. Thus, they might be exposed to the disease unintentionally.

Despite the association between Rh blood group and HBV infection is conflicting, Rh-negative women had more likely to develop HBV infection than Rh-positives in the present study. This finding was in agreement with previous evidence of Côte D’Ivoire and China [44, 45]. The statistical association of Rh blood group with HBV infection might be due to the interaction between membranes of red blood cells (RBC) even though host susceptibility related with Rh blood group has not yet been clarified [46, 47]. Moreover, the same finding was observed in Rh-negative women to develop syphilis infection. The relationship between viral hepatitis as well as syphilis and Rh blood group needs to be addressed in future studies.

The practical implication of this study is that, the high burden of STIs among pregnant women will lead to unwanted pregnancy outcomes such as increased neonatal mortality rate, still birth, vertical transmission, preterm delivery as well as low birth weight and psychological problems. Moreover, the finding of this study will be used for enforcing the policymakers to revise the currently implementing National Reproductive Health Strategy of Health Sector Transformation Plan II of the country which targets reproductive health Problems including STIs and working to improve the health care coverage in Ethiopia. However, as a limitation the prevalence of hepatitis virus infection particularly HBV infection might be underestimated because occult HBV infection was not detected due to the absence of diagnostic facilities such as quantitative real time polymerase chain reaction (RT-PCR).

## 5. Conclusion

The magnitude of HBV and HCV infection was clustered as high and moderate, respectively. The magnitude of overlapping and parallel infections of HIV, syphilis, HBV and HCV still remained one of the significant public health problems in our study area requiring a strong multi-sectoral effort to tackle the transmission among key populations. The high frequency of overlapping infection of HBV-HIV urges the policy makers and other concerned bodies or officials to initiate HBV virus screening for HIV/AIDS patients to prevent clinical and therapeutic interactions of the diseases. Increasing age, RH positivity, rural residence, low level of education, tattooing, multiple sexual partners and exposure to unsafe sex were identified significant risk factors for STIs. Therefore, aggressive reproductive health education campaigns and health education or information education communication about STIs are highly advisable to bring behaviour change about the risk factors, transmission and prevention of STIs as well as the importance of ANC follow-up in early pregnancy. Moreover, continuous screening of pregnant women for HIV, syphilis, hepatitis B and C infections should be performed and special attention should be given for pregnant women who had co-infections.

## Supporting information

S1 Data (CSV)

## Abbreviations

ANC: Antenatal Care
AOR: Adjusted Odds Ratio
HBV: Hepatitis B Virus
HCV: Hepatitis C Virus
HIV: Human Immunodeficiency Virus
RH: Rhesus
STI: Sexually Transmitted Infections
